# Olanzapine- induced metabolic syndrome pathogenesis: Hypothalamic “leptin resistance” or “pomc resistance”?

**DOI:** 10.1192/j.eurpsy.2021.408

**Published:** 2021-08-13

**Authors:** F. Veneziani, M. Zezza, A. Marakhovskaia, J.M. Beaulieu

**Affiliations:** 1 Pharmacology And Toxicology, University of Toronto, Toronto, Canada; 2 School Of Medicine And Surgery, University of Bari, Bari, Italy

**Keywords:** Translational medicine, Metabolic syndrome, Hypothalamic RNAseq analysis, Psychopharmacology

## Abstract

**Introduction:**

Olanzapine (OL) represents one of the main chooses for the treatment of psychosis. However, OL increase the risk of metabolic syndrome (MS). Previous literature proposes the “Leptin resistance” as a possible hypothalamic pathogenesis of OL induced MS because of the occurrence of weight gain with increased Leptin blood level.

**Objectives:**

The aim of our study is to investigate in a murine model of full-MS phenotype induced by Olanzapine the hypothalamic gene expression of the pathways involved in Leptin receptor signalling to clarify if a Leptin resistance occurs.

**Methods:**

For the experiment C57BL/6Jfemale mice are used.The OL group (n=15) received pure Olanzapine compounded into chow (54 mg/kg of diet). The vehicle group (n=15)is fed with the same chow without OL. Weight gain is measured every 5 days. After 4 weeks of treatment, mice are sacrificed by rapid cervical dislocation. Blood is collected for Glucose, Insulin and Leptin evaluation. Hypothalamus is dissected and RNA-seq is performed.

**Results:**

The OL group shows a significantweight gain compared to Control (p= 0.02). Likewise blood glucose, Insulin and Leptin levels appear increased (p= 0,0089, p= 0,01, p= 0,0012). From the analysis of RNA-seq hypothalamic differentially expressed genes the anorexigenic POMC pathway downstream to the Leptin Receptor shows a 4-fold increased compared to control.

**Conclusions:**

In our study the “Leptin resistance” involvement in OL induced MS is not confirmed. In fact, although there is an increase in blood Leptin, the expression of Leptin receptor downstream pathways shows a significant increase. This could suggest a possible “POMC resistance” mechanism for OL induced MS.

**Disclosure:**

No significant relationships.

